# Screening for diabetes and hypertension in a rural low income setting in western Kenya utilizing home-based and community-based strategies

**DOI:** 10.1186/1744-8603-9-21

**Published:** 2013-05-16

**Authors:** Sonak D Pastakia, Shamim M Ali, Jemima H Kamano, Constantine O Akwanalo, Samson K Ndege, Victor L Buckwalter, Rajesh Vedanthan, Gerald S Bloomfield

**Affiliations:** 1 Purdue University College of Pharmacy, W7555 Myers Building, 1001 W. 10th Street, Indianapolis, Indiana, USA; 2 Department of Medicine, School of Medicine, College of Health Science, Moi University, Eldoret, Kenya; 3 Moi Teaching and Referral Hospital, Nandi Road, Po Box 3, Eldoret, Kenya; 4 Academic Model Providing Access to Healthcare (AMPATH), Nandi Road, PO Box 4606, Eldoret, Kenya; 5 Division of Cardiovascular Medicine, Duke University Medical Center and Duke Clinical Research Institute, North Pavilion, 2400 Pratt Street, Durham, NC, USA; 6 Department of Epidemiology and Nutrition, School of Public Health, College of Health Sciences, Moi University, Nandi Road, PO Box 4606, Eldoret, Kenya; 7 Webuye District Hospital, Moi Street, PO Box 25, Webuye, Kenya; 8 Department of Family Medicine, School of Medicine, College of Health Science, Moi University, Nandi Road, PO Box 4606, Eldoret, Kenya; 9 Zena and Michael A. Wiener Cardiovascular Institute, Icahn School of Medicine at Mount Sinai, One Gustave L. Levy Place, New York City, NY, USA

**Keywords:** Diabetes, Hypertension, Kenya, Cardiovascular disease, Home-based screening, Community-based screening

## Abstract

**Background:**

The burdens of hypertension and diabetes are increasing in low- and middle-income countries (LMICs). It is important to identify patients with these conditions early in the disease process. The goal of this study, therefore, is to compare community- versus home-based screening for hypertension and diabetes in Kenya.

**Methods:**

This was a feasibility study conducted by the Academic Model Providing Access to Healthcare (AMPATH) program in Webuye, a town in western Kenya. Home-based (door-to-door) screening occurred in March 2010 and community-based screening in November 2011. HIV counselors were trained to screen for diabetes and hypertension in the home-based screening with local district hospital based staff conducting the community-based screening. Participants >18 years old qualified for screening in both groups. Counselors referred all participants with a systolic blood pressure (SBP) ≥160 mmHg and/or a random blood glucose ≥7 mmol/L (126 mg/dL) to a local clinic for follow-up. Differences in likelihood of screening positive between the two strategies were compared using Fischer’s Exact Test. Logistic regression models were used to identify factors associated with the likelihood of following-up after a positive screening.

**Results:**

There were 236 participants in home-based screening: 13 (6%) had a SBP ≥160 mmHg, and 54 (23%) had a random glucose ≥ 7 mmol/L. There were 346 participants in community-based screening: 35 (10%) had a SBP ≥160 mmHg, and 27 (8%) had a random glucose ≥ 7 mmol/L. Participants in community-based screening were twice as likely to screen positive for hypertension compared to home-based screening (OR=1.93, *P*=0.06). In contrast, participants were 3.5 times more likely to screen positive for a random blood glucose ≥7 mmol/L with home-based screening (OR=3.51, *P*<0.01). Rates for following-up at the clinic after a positive screen were low for both groups with 31% of patients with an elevated SBP returning for confirmation in both the community-based and home-based group (*P*=1.0). Follow-up after a random glucose was also low with 23% returning in the home-based group and 22% in the community-based group (*P*=1.0).

**Conclusion:**

Community- or home-based screening for diabetes and hypertension in LMICs is feasible. Due to low rates of follow-up, screening efforts in rural settings should focus on linking cases to care.

## Background

In Kenya, hypertension and diabetes mellitus are considered widespread problems but there are few studies reporting the prevalence of these diseases or replicable screening strategies. An analysis of worldwide data in 2005 showed that 639 million (625–654 million) patients with hypertension live in low and middle income countries (LMICs) [1,2]. By 2025, the number of adults with hypertension is predicted to increase by about 60% and almost three-quarters of the world’s hypertensive population will live in LMICs [3]. Studies have also found similar trends in the prevalence of diabetes, with prevalence rates ranging from < 1% in rural areas to > 20% in urban settings with variation according to racial/ethnic group [4]. The International Diabetes Federation estimates that the number of individuals with diabetes in Africa will double to 23.9 million people by the year 2030 [5,6].

In Kenya, the available data on the burden of hypertension or diabetes suggests prevalence rates of 12% and 6.6%, respectively [7]. However, low awareness of chronic diseases, poverty, and health system factors, among other issues, may lead to underestimates of the true prevalence [8]. While worldwide rates of diabetes and hypertension appear to be increasing, the paucity of locally relevant data can hinder planning and developing preventative and clinical care strategies to manage these diseases. As such, strategies to improve the availability and accuracy of local prevalence data are needed.

Whether home-based or community-based screening is more appropriate in LMICs such as Kenya is unknown. We therefore conducted a feasibility study to compare a community-based versus home-based screening strategy for hypertension and diabetes. To assess the feasibility of these approaches, our primary aim was to obtain an estimate of disease prevalence and describe the populations using both approaches. We also explored the pattern of referral using various thresholds levels for blood pressure and blood sugar measured during the screening exercise. We hope to use these comparative assessments to inform local public health policy and health system planning for future screening activities.

## Methods

### Study setting

This pilot study is an initiative of the Academic Model Providing Access to Healthcare (AMPATH) program located in western Kenya, which is a partnership between Moi University, Moi Teaching and Referral Hospital and a consortium of North American universities led by the Indiana University School of Medicine. The history, organizational structure, and health programs of AMPATH have been described elsewhere [9-11]. AMPATH has the stated goal to build upon an academic foundation that supports all 3 academic missions of service (through healthcare), teaching, and research. AMPATH delivers care, provides education, and performs research in networks of urban and rural Ministry of Health hospitals, health centers, and dispensaries in western Kenya. AMPATH has also developed considerable infrastructure for providing home-based and community-based counseling and testing for HIV and has pioneered several innovative strategies for integrating intensified case finding into its comprehensive prevention strategy [9,12]. AMPATH delivers a comprehensive, community-based care program that initially focused on patients infected with HIV but has since expanded to provide maternal and child health services and chronic disease management (specifically diabetes and hypertension) to a catchment population of over 2 million persons [2]. This specific feasibility testing of screening for hypertension and diabetes was carried out in the rural town of Webuye which is located within the AMPATH catchment area. Webuye town (population 19,600 in 2011) was selected to carry out the pilot because home-based counseling and testing (HBCT) for HIV was underway at the time of the study [9,12]. There also exists accessible infrastructure for long-term care of diabetes and hypertension at Webuye District Hospital to manage all the positively screened patients [13]. This project was approved by the Institutional Research and Ethics Committee based at Moi University School of Medicine.

### Home-based screening

The home-based screening pilot was carried out in March 2010 alongside the ongoing HBCT for HIV. Five home-based counselors (trained in HIV testing and counseling) with at least a high school education who were contemporaneously providing home-based HIV screening underwent a one-day training session on hypertension and diabetes. This training highlighted the epidemiology, pathophysiology, risk factors, diagnosis/measurement and screening methods for hypertension and diabetes. Counselors were also instructed on the overall goals of treatment and prevention options. Lastly, in practical sessions, the counselors were taught about the appropriate methodology for performing finger pricks, proper use of glucose testing strips and meters (Abbott Optimum Xceed), and proper use of an automatic sphygmomanometer (OMRON HEM-712c) with a medium sized cuff (22 to 32 cm). Each counselor was instructed, observed and required to demonstrate proper diabetes and hypertension screening techniques. After completion of the training session, investigators (SP, SMA, COA, GSB) accompanied counselors into at least one household to ensure that they were able to provide appropriate counseling and to measure blood pressure and blood sugar using the proper technique.

All individuals 18 years of age and above who verbally consented to have their blood pressure and sugar measured were eligible. Counselors traveled door-to-door to offer screening services using a standardized protocol. After receiving verbal consent from the participants, counselors counseled and tested participants for HIV. Participants were also counseled on hypertension and diabetes screening and subsequently tested for random blood sugar (RBS). Blood pressure was measured at the end of the home visit after the counselor had tested for blood sugar and HIV. Blood pressure was measured only once unless an error message was recorded. In the case of an error message, most participants had their blood pressure measured again. If an error message was persistently observed, participants were instructed to follow up at the specified confirmation site at Webuye District Hospital.

### Community-based screening

The community-based screening strategy was carried out in November 2011 in conjunction with Webuye District Hospital staff. Community mobilizers were used to sensitize the community to the availability of a 2-day long diabetes and hypertension screening program in the upcoming week to commemorate World Diabetes Day. The availability of this free screening was advertised through standard modalities of sensitization including discussion at church, via community chiefs meetings (locally referred to as *Barazas*), and by word of mouth via community leaders. Any person 18 years of age and above voluntarily visiting the screening booth received a free blood pressure check, free blood sugar test, had their height and weight recorded, and BMI calculated. The screening booth was situated in the center of town and was marked with signage and staff who actively advertised for the screening. The screening booth was easily visible and accessible to any participant interested in a free screening. Nurses and clinical staff with experience in the management of chronic diseases at the Webuye District Hospital chronic disease clinics were responsible for performing all elements of the screening program.

### Screening and referral protocol

The same screening and referral protocol was used for both the home-based and community-based screening strategies. Age, sex, medical record number (if available), contact information, HIV screening result (only performed in home-based screening), blood pressure screening result, and blood sugar screening result were recorded manually for all participants by the counselors on a standardized data collection sheet. In the community-based screening event, screening staff also recorded the height and weight on the same data collection sheet.

A cut-off systolic blood pressure (SBP) of ≥ 160 mmHg was used in order to triage screened participants for referral to the local clinic for diagnostic testing. This cut-off was based on the limitation that only one blood pressure reading was obtained during screening and to avoid unnecessary referral due to one isolated high reading and regression towards the mean. In addition, previous studies in sub-Saharan Africa have used a higher screening threshold to avoid excess referral in settings with resource constraints to maximize the use of resources [14-18]. A diastolic blood pressure (DBP) cut-off was not used to screen participants [19]. Participants who met referral criteria were referred to the local clinic located at Webuye District Hospital for follow-up blood pressure measurements. At that follow-up visit, two blood pressure measurements were taken during the same visit. The average of the two blood pressures taken at the clinic was calculated. Hypertension was diagnosed based on the Joint National Committee VII criteria for systolic or diastolic blood pressure (SBP ≥ 140 or DBP ≥ 90 mmHg) [20].

A RBS cut-off of 7.0 mmol/L (126 mg/dL) was used as the threshold for referring participants for confirmatory testing in clinic. With the unpredictable nature of the timing of the screening, it is possible that some participants would have been fasting at the time of screening. Therefore, the recommended RBS cutoff of 11.1 mmol/L (200 mg/dL) for diagnosing diabetes would not have appropriately referred participants who arrived to the screening with a fasting blood sugar result ≥ 7.0 mmol/L. Because of this dynamic, the threshold for referral for all participants was set at 7.0 mmol/L to ensure that any patient who might meet either of these diagnostic criteria would be offered confirmatory testing. Patients meeting this referral threshold were then instructed to fast before coming for confirmatory testing at the hospital based outpatient diabetes clinic on a subsequent day. Patients with a fasting blood sugar above 7.0 mmol/L in the clinic were confirmed to have diabetes.

Participants with a positive screen for hypertension (systolic blood pressure ≥ 160 mmHg) or diabetes (RBS ≥ 7 mmol/L) were provided an information sheet and referral card to follow up in clinic at the Webuye District Hospital. The participants who returned to the hospital based clinic were then provided with a free fasting blood sugar testing and/or 2 separate resting blood pressure readings to confirm the relevant diagnosis.

Participants found to have SBP 140–159 mmHg during the initial screening were provided education on appropriate lifestyle modifications and dietary strategies, such as salt reduction. They were also instructed to obtain a follow-up blood pressure reading within 6 months at any local blood pressure testing facility. Participants with impaired fasting glucose (5.6 – 6.9 mmol/L or 100 - 125 mg/dL) on the clinic based confirmatory fasting blood glucose testing were also instructed to engage in lifestyle modifications and perform an annual fasting blood sugar at the nearest available facility.

All participants receiving a confirmed diagnosis of diabetes or hypertension were instructed to engage in lifestyle modifications and were registered into the appropriate chronic disease clinic based on their diagnosis.

Participants who did not follow up after having a positive screen for diabetes or hypertension in the initial screening were contacted via phone and provided additional directions and encouragement to visit Webuye District Hospital for confirmation. Participants were called on at least two separate occasions at both the primary and alternate phone number provided during the respective screening events.

### Statistical analysis

All participants with complete data recorded in the screening register were included in this analysis. Descriptive analyses were used to characterize the demographic characteristics of the findings of the two different screening strategies. To achieve the primary objective of comparing the feasibility of both strategies, descriptive analyses were used to assess the percentage of participants with an initial positive screen for diabetes and elevated SBP in both strategies. In addition, comparative statistical analyses were performed to identify statistically significant differences between the two strategies for diabetes and hypertension screening. The Fischer’s Exact Test was utilized to compare the difference in the likelihood of screening positive for diabetes or hypertension in the home-based versus community-based screening strategy. Additionally, the likelihood of following up after a positive screening test was compared between the two screening strategies and odds ratios (OR) for a positive screening were calculated. Exact logistic regression was performed to determine the characteristics associated with a positive screening for either diabetes or hypertension. Linear regression was utilized to identify the relationship of relevant covariates with blood pressure and blood sugar. In order to demonstrate the changes that might occur by setting more aggressive referral thresholds, additional analyses were completed to illustrate the additional numbers of patients that might be referred using different screening criteria. All analyses were completed using STATA® (College Station, Texas, Version 8).

## Results

### Overall findings

**Figure 1:**
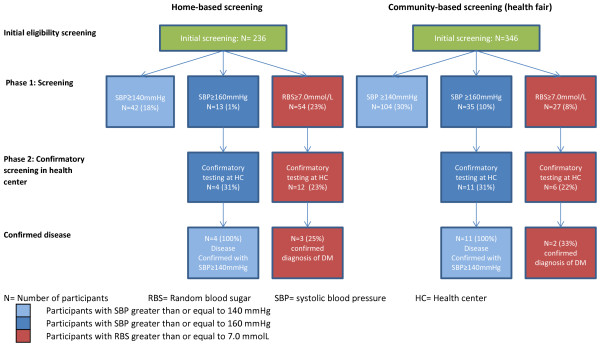
Flow chart for screening results in home- and community-based screening.

There were 236 participants in the home-based screening and 346 participants in the community-based screening who met the inclusion criteria for this analysis with participants having a mean age of 37 (SD=15) and 39 (SD=13) years, respectively (Figure 1). As seen in Table 1, the home-based screening strategy identified 13 participants (6% of the total population screened) with a SBP greater than or equal to 160 mmHg, while the community-based strategy identified 35 participants (10% of the total population screened). Participants in the community-based screening were almost twice as likely to have a positive screening for hypertension compared to the home-based screening arm (OR=1.93, Fischer’s exact test, *P*=0.06). With regards to diabetes screening, 54 participants (23%) and 27 participants (8%) in the home-based screening and community-based screening, respectively, met the predefined threshold requiring confirmatory blood sugar testing. Participants in the home-based screening were 3.5 times more likely to have a positive screening result than the participants in the community-based screening (OR=3.51, Fischer’s exact test, *P*<0.01).

**Table 1:** Characteristics of the home-based and community-based screening program for diabetes and hypertension

	**Home-based screening**	**Community-based screening**
Number Screened	236	346
Age category (years)		
18-24, n (%)	48 (20%)	49 (14%)
25-34	88 (37%)	99 (29%)
35-44	39 (17%)	85 (25%)
45-54	28 (12%)	69 (20%)
55-64	21 (9%)	32 (9%)
65-74	6 (3%)	12 (3%)
≥75	6 (3%)	0
Female, n (%)	146 (61%)	204 (59%)
SBP, mean (SD) mmHg	125 (19.28)	134 (21.35)
<120, n(%)	111 (47%)	85 (25%)
120 – 139	83 (35%)	157 (45%)
140 – 159	29 (12%)	69 (20%)
160- 179	10 (4%)	20 (6%)
180 – 199	2 (1%)	10 (3%)
≥200	1 (1%)	5 (1%)
DBP, mean (SD) mmHg	77 (13)	78 (11.34)
<60, n (%)	8 (3%)	15 (4%)
60 – 79	134 (57%)	203 (59%)
80 – 89	58 (25%)	74 (21%)
90 – 99	26 (11%)	40 (12%)
≥100	10 (4%)	14(4%)
RBS, mean (SD) mmol/L	6.1 (2)	5.0 (1.8)
<7.0, n (%)	182 (77%)	319 (92%)
7.0 - 9.9	48 (20%)	21 (6%)
10.0 - 12.9	4 (2%)	3 (1%)
≥ 13.0	2 (1%)	3 (1%)
Follow-up for Hypertension/Diabetes		
Number of participants with elevated systolic blood pressures above 160 mmHg returning for confirmation	4 (31% of participants with an elevated reading)	11 (31% of participants with an elevated reading)
Number of participants with elevated systolic blood pressures above 160 mmHg returning for follow-up care	3	11
Number of participants with random blood sugars above 7 mmol/L returning for confirmation	12 (23% of participants with an elevated reading)	6 (22% of participants with an elevated reading)
Number of participants with elevated blood sugars confirmed to have diabetes and enrolled for follow-up care	3	2
Number of participants with both overt diabetes (RBS>9.9 mmol/L) and hypertension (SBP>160 mmHg) on initial screening	3	2
BMI, mean (SD) kg/m^2^		23.0 (3.8)
<16.5		3
16.5-18.4		19
18.5-24.9		244
25-29.9		61
30-34.9		15
35-40		4

*SD*=standard deviation, *SBP*=systolic blood pressure, *DBP*=diastolic blood pressure, *RBS*=random blood sugar, *BP*= blood pressure, *SBP*=systolic blood pressure, *BMI*= body mass index, *kg*=kilograms, *m*=meters.

#### Follow-up rates

The rate of follow-up for confirmatory testing in clinic was low for both screening strategies. Of the 13 participants in the home-based screening arm with a SBP ≥160 mmHg, 4 (31%) returned for confirmation. All 4 of these participants had a SBP ≥ 140 mmHg when confirmatory testing was performed, thereby meeting criteria for the confirmatory diagnosis of hypertension. Similarly, in the community-based screening, 11 (31%) of the 35 participants identified with an SBP ≥ 160 mmHg returned for confirmation and all of these participants were confirmed to have a SBP ≥ 140 mmHg. There was no difference in the odds of returning for confirmatory follow up between the two groups (OR=0.97, Fischer’s exact test, *P*=1.00).

In the analysis of blood sugar screening, 12 (23%) of the 54 participants meeting the predefined criteria for referral returned for the confirmatory fasting blood sugar in the home-based screening with 3 receiving a confirmed diagnosis of diabetes mellitus. In the community-based arm, 6 (22%) of the 27 participants returned for the confirmatory fasting blood sugar with only 2 participants being confirmatively diagnosed with diabetes. There was no significant difference in the odds of returning for confirmatory follow up between the two groups (OR=1.00, Fischer’s exact test, *P*=1.00). With the limited number of participants returning for the fasting blood confirmation and the limited utility of a single random blood sugar, secondary analysis did not reveal any meaningful results beyond the low follow-up rate. None of the participants in the home-based screening who had elevated blood pressures or blood sugars tested positive for HIV. An informal survey of the counselors at the end of home-based screening revealed that participants who were initially reluctant to undergo HIV testing were more likely to be tested if they were offered screening for diabetes or hypertension at the same time.

#### Alternate referral thresholds

If the threshold for referral during screening was set to a SBP of ≥ 140 mmHg, an additional 29 participants (12%) and 69 participants (20%) in the home-based and community-based screening program, respectively, would have been referred for confirmatory testing. Adding a DBP cutoff of ≥ 90 mmHg alongside SBP ≥ 140 mHg as a threshold for referral would have resulted in an additional 4 participants in the community basing screening and 16 in the home-based screening requiring referral. If a DBP ≥ 100 mmHg was included, 4 participants and 2 additional participants in the home-based and community-based screening, respectively, would have been referred for confirmatory testing assuming that all participants with a SBP ≥ 160 mmHg would have already been referred.

In the combined analysis of the screening strategies, there was a statistically significant higher likelihood of screening positive for an elevated SBP if they screened positive for an elevated RBS (*P* < 0.01). Likewise, there was a statistically significantly higher likelihood of screening positive for an elevated RBS if they screened positive for an elevated SBP (*P* < 0.01).

In the secondary analysis, univariate and multivariate linear regression analysis revealed that participants of older age were more likely to have a higher SBP (*P* < 0.01) in the combined analysis of the home and community-based screening with screen-positive participants having a mean age of 52 compared to a mean age of 37 for screen-negative participants. Other demographic characteristics including gender and BMI (only evaluated in community-based screening) did not have statistically significant associations with SBP.

## Discussion

Hypertension and diabetes mellitus are two increasingly common conditions in LMICs that expose patients to increased risk of mortality and morbidity [21]. Identifying participants in the pre-clinical stages by screening offers participants and providers an opportunity to modify long-term risk before serious complications occur [22]. By performing both home-based and community-based screening pilot studies for hypertension and diabetes mellitus in western Kenya, we have gained important insight into the burden of these conditions, challenges in the long-term care of patients with these conditions and the comparative advantages and disadvantages of both strategies.

With a SBP screening referral cutpoint of 160 mmHg, the home-based screening found 6% of the population screened positive compared to 10% in the community-based screening arm. While other studies have found prevalence rates as high as 50% in Kenya, certain key differences in methodology must be considered when assessing this information [7]. In this study, a higher blood pressure threshold was set and the population being screened was predominantly rural with a distinct tribal constituency largely made up of the Bukusu tribe. The low prevalence of confirmed diabetes (1.2% in HBCT versus 0.6% in the community-based arm) must also be interpreted cautiously when comparing the findings to other studies as this study utilized a more contextualized screening strategy designed to fit within AMPATH’s overarching screening approach while not necessarily following all the standard diagnostic criteria typically utizlized in resource-rich settings. A consistent finding between the two strategies was the low rate of follow-up amongst patients who met the screening threshold. Despite the inclusion of a system for phone-based reminders for all participants with elevated results, the follow-up rate for confirmatory testing was low and many participants were unavailable via the phone number provided or unwilling to come in to the referral hospital. This lack of follow-up illustrates a major deficiency in the feasibility of a one-time screening approach amongst rural patient populations without an intensive linkage strategy to ensure patients enroll into a care program. Patient education and limited awareness of the need for testing, cost of travel or other factors may also have been responsible for the low follow-up rate despite the provision of education by community health workers and relatively close proximity of the health facility to patients screened (90% of screened patients lived within 2 miles of the facility). This investigation also highlights the need for confirmatory screening strategies that can be completed in one encounter in either the home- or community-based setting. For example, as additional data emerges for the role of glycosylated hemoglobin (A1c) in diagnosing diabetes in African populations, this testing strategy can be utilized to provide point-of-care confirmation in one encounter with or without prior screening. Currently, this approach is largely limited by the excessive costs of this form of testing, limited data on the reliability of A1c in sub-Saharan African populations, and the resource constraints which pervade this setting. With the limited interpretation which can come from a single RBS and difficulty in obtaining a fasting blood sugar in a voluntary impromptu screening, there is considerable need for more contextualized strategies [23]. As access to point of care A1c testing is expanded in resource-constrained settings, it is hoped that questions about its accuracy in these populations can be addressed and subsequently be integrated into home-based screening and care to continue to improve the ease with which patients can be screened to confirm diabetes [24,25].

The study utilized a voluntary, convenience sampling approach to screen a broad range of patients regardless of their baseline risks for diabetes or hypertension. The inclusive nature of this screening program and low follow-up rates for confirmatory testing are largely responsible for the relatively low prevalence rates observed during this screening activity. Patients voluntarily submitting to screening in the community-based arm were more likely to screen positive for elevated SBP than in the home-based arm. Because of the voluntary nature of this screening, it is possible that patients with a higher risk or family history of hypertension were more likely to submit to screening than in the home-based arm which screened all eligible patients in the homes visited. Based on these findings, more targeted screenings assessing high-risk populations will be conducted to focus on the delivery of care to patients more likely to have hypertension. Conversely, a higher percentage of patients met the positive screening threshold for diabetes in the home-based arm than in the community-based arm. One potential explanation for this is that many participants were screened shortly after having their morning or afternoon tea. It is possible the timing of the consumption of sugar containing tea and the completion of a random blood glucose screening could have led to a small increase in their blood glucose measurement above 7.0 mmol/L as 20% of the patients in the home-based arm compared to 6% in the community-based arm had a RBS in the 7.0 – 9.9 mmol/L range. Because of the low rates of follow-up, it is unclear whether these mildly elevated random blood glucoses represent true cases of diabetes or false positive screenings.

Since completing this investigation, AMPATH has partnered with the Kenyan Ministry of Health to begin wide scale implementation of a diabetes and hypertension screening program. To mitigate against low rates of follow-up, a linkage strategy including the provision of home-based care via community health workers has been incorporated. This integrated approach utilizing governmental partnership has been a vital component to addressing the large healthcare workforce needs for chronic disease management. As demonstrated in this study, setting a cut point of referral for participants with a SBP of greater than 160 mmHg results in a smaller number of people who will be referred for confirmation compared to lower SBP thresholds. In addition, we did not include a DBP cut-off yet found many participants with single DBP measurements greater than or equal to 90 mmHg. Whether lower thresholds would have resulted in a higher or lower true positive rate is unknown given the low numbers of participants who returned for follow-up and that those with lower SBPs or elevated DBP were not referred for testing. However, with the availability of a larger pool of healthcare workers through partnership with the Ministry of Health, participants are now being referred if they have a SBP ≥ 140 mmHg or DBP ≥ 90 mmHg in order to ensure that confirmatory testing is offered to the greatest number of persons. With the limited number of confirmed cases of diabetes and lack of follow up after elevated RBS, it is difficult to utilize this feasibility assessment to refine the cutoff values for diabetes referral. To better understand the optimal cutoff values, we are currently employing a revised strategy in which patients are scheduled to have confirmatory testing for fasting blood sugar via home-based testing or at a local public health facility that is within close proximity to their residence.

While the primary goal of this study was to demonstrate feasibility through practical implementation, there are several key limitations associated with this approach. Because of the logistical challenges in implementing the different screening programs, there was a considerable delay in the time frames that both strategies were implemented. Because of this limitation, it is possible that there was some overlap in the patients participating in both screening activities. The potential also exists for an increase in the awareness and prevalence of chronic diseases during the time between the two screening strategies which could confound the comparison of the strategies.

One of the challenges in implementing a contextualized program for this pilot was setting appropriate thresholds for referral for diabetes and hypertension. Because of the convenience sampling approach, we were only able to perform a single screening BP and random blood screening. While neither approach represents the recommended diagnostic approach in isolation, it represents the most practical screening approach given the limitations of the rural setting in which this pilot was conducted. Through the anecdotal feedback from the screening counselors, intermittent error readings were found when performing blood pressure measurements. However, after performing repeat tests, they were able to obtain blood pressure readings on all the participants at the time of the home-based or community based screening. As we continue to expand the chronic disease management program to additional areas we hope to continue to revise our referral thresholds and screening approaches to ensure we maximize the long-term health care benefits for the populations we serve within the limited resources available. In addition, the potential impact of offering screening for hypertension or diabetes alongside HIV testing on acceptability of HIV testing needs to be explored further. Despite the challenges, we were able to assess the different screening strategies and identify the primary barriers with each strategy. This activity has greatly assisted our ongoing efforts to build sustainable chronic disease infrastructure throughout our western Kenyan catchment area.

With the logistical challenges of providing laboratory-based diagnosis of diabetes using venous blood samples, this study relied on the less preferred diagnostic strategy based on point-of-care glucose meters. While these have not been formally approved for diagnosis, they have been suggested as a suitable alternate testing strategy in settings where laboratory based diagnosis is not readily available [26].

## Conclusions

This study illustrates that home- and community-based screening for hypertension and diabetes can be carried out in Kenya’s rural areas. This investigation also illustrates the feasibility of screening for chronic diseases alongside the infrastructure that has been built to address the multifactorial aspects of HIV management and prevention. Both screening strategies identified a large pool of high-risk participants which had similar rates of poor follow-up after screening. When screening for hypertension is assessed in isolation from the other aspects of the healthcare system, the community-based screening strategy seems to attract higher risk participants compared to a more comprehensive home-based screening strategy. One of the major benefits of home-based screening, not specifically discussed in this study, is the potential for greater linkage to the healthcare system to facilitate home-based care in the future.

Based on the observations in this investigation the decision to utilize a community-based versus home-based should largely be based on the overarching capacity of the healthcare system in which it is being implemented. For settings where considerable infrastructure exists for more portable, home-based care and diagnosis, the home-based screening strategy provides many potential advantages in the long-term management of patients beyond screening. For example, within AMPATH, smartphones are now used to capture the GPS coordinates of citizens within our catchment area for subsequent registration and follow-up from community healthcare workers [12]. For settings where care is largely centralized at healthcare facilities, community-based screening provides a quick and easy to implement approach with a potentially higher yield of high risk patients.

However, to increase the long-term health benefits for the rural populations these efforts are intended to serve, an integrated approach linking screening, care and follow-up regardless of the point of service delivery needs to be implemented to reduce the preventable complications of hypertension and diabetes.

## Competing interest

Sonak Pastakia has received fees for serving as a speaker and consultant for Abbott within the last three years.

## Authors’ contributions

SDP oversaw the entire project including conception, implementation, and drafting of the initial manuscript. SMA assisted with the implementation of the project and manuscript preparation. JK helped design the project and assisted with manuscript preparation. COA assisted with the implementation of the project and assisted in manuscript preparation. SKN assisted with the implementation of the project and manuscript preparation. VB assisted with the design and implementation of the project in addition to the manuscript preparation. RV assisted with design of the analysis and manuscript preparation. GSB oversaw the entire project including conception, implementation, and manuscript preparation. All authors read and approved the final manuscript.
